# The role of individual and community factors on institutional delivery in Somaliland: a study based on the 2020 Somaliland demographic health survey

**DOI:** 10.3389/fgwh.2025.1574471

**Published:** 2025-10-07

**Authors:** Hamze G. Dahir, Hodo Abdikarim, Hibo Abdirashid, Hafsa Mohamed, Abdisalam Hassan Muse, Abdirashid M. Yousuf, Mohamed A. Hussein

**Affiliations:** 1 School of Public Health and Nutrition, College of Health Science, Amoud University, Borama, Somaliland; 2 Faculty of Science and Humanities, School of Postgraduate Studies and Research (SPGSR), Amoud University, Borama, Somaliland; 3 Research and Innovation Center, Amoud University, Borama, Somaliland

**Keywords:** institutional delivery, maternal health, multilevel analysis, socio-economic factors, Somaliland

## Abstract

**Background:**

Maternal mortality remains a critical concern in low-income countries, where low utilization of institutional delivery services is a contributing factor. This study investigates the influence of individual and community-level factors on institutional delivery among women in Somaliland, a region with a high maternal mortality rate.

**Methods:**

This cross-sectional study used data from the 2020 Somaliland Demographic and Health Survey (SDHS), a nationally representative survey of 3804 women aged 15–49. We employed descriptive statistics and Chi-square tests to examine bivariate associations and multi-level binary logistic regression to assess the impact of individual and community-level factors on the place of delivery.

**Results:**

Only 30.8% of deliveries occurred in health institutions. Bivariate analyses showed significant associations between institutional delivery and maternal age, education (*χ*² = 328.534, *p* < 0.001), husband's education (*χ*² = 362.669, *p* < 0.001), wealth (*χ*² = 787.937, *p* < 0.001), region (*χ*² = 50.760, *p* < 0.001), and parity (*χ*² = 65.227, *p* < 0.001). Multilevel analysis revealed that 50% of the variance in place of delivery was attributable to community level factors (Model I). Higher maternal education was significantly associated with increased odds of institutional delivery (AOR = 8.87, *p* < 0.05), while nomadic residence (AOR = 0.28, *p* < 0.05), residing in Sanaag region (AOR = 0.36, *p* < 0.05), high parity (five or more children) (AOR = 0.52, *p* < 0.05), not intending to use contraceptives (AOR = 0.62, *p* < 0.05), and wanting the pregnancy later (AOR = 0.79, *p* < 0.05) were significantly associated with decreased odds of institutional delivery. Women in the highest wealth quintile were significantly more likely to deliver in a health facility (AOR = 18.71, *p* < 0.05).

**Conclusion:**

The study highlights the complex interplay of individual and community-level factors influencing the utilization of institutional delivery in Somaliland. Interventions to promote health facility deliveries must address socioeconomic disparities, improve women's education, ensure accessibility of healthcare for nomadic communities, reduce regional variations, promote family planning and reproductive health services, and take into consideration the impact of parity on health seeking behaviors.

## Introduction

Utilization of prenatal care (ANC), trained birth attendants, and postnatal care are among the important obstetric care services that are utilized to help reduce maternal and newborn death and morbidity in low-income countries (1, 2). Different birthing practices exist around the world, based on the cultural setting of each society. In some places, ladies give birth to their children without anyone watching. A few look for a midwife and an obstetrician, although traditional birth attendants who lack training help with two-thirds of births worldwide (3).

An indicator of the reduction of maternal mortality is the delivery by expert birth attendance (4). The place of delivery has a significant impact on both the mother's and the newborn's health and well-being. When mothers give birth to a child they desire, it's typically a happy moment. Both the mother's and the baby's health are at risk during the birthing process. If issues are not appropriately and successfully addressed during labor and delivery, one or both of them may become unwell or possibly die (3, 5).

The number of women who died globally in 2020 from pregnancy and childbirth was approximately 295,000, which is unacceptable, even though there has been a lot of progress in the last 20 years. 2020 saw about 95% of maternal deaths take place in low-income and lower-middle-income nations (6, 7). Maternal mortality rates in Southern Asia and sub-Saharan Africa make up 86% of all deaths globally (8).

Studies show that in low-resource African countries, institutional delivery also referred to as giving birth outside of a medical facility is often the cause of maternal deaths (9, 10). Women who reside in rural areas, travel great distances to medical institutions, are from low-income backgrounds, have restricted access to birthing facilities, and have inadequate mechanisms in place for referring women experiencing obstetric emergencies are additional factors (11–14).

Maintaining the availability and accessibility of professional care during pregnancy and childbirth is a top priority for the Safe Motherhood Initiative, and institutional delivery is seen as an essential part of this endeavor (15, 16). Within any country's health plan, the proportion of women who receive expert assistance during birth is an important indication. It has been determined that one of the most important methods for reducing maternal mortality is institutional delivery (17–19). The frequency of preventable maternal and infant deaths decreases when a woman gives birth in a medical facility where she may receive the required care during the giving procedure (19–21).

The utilization of competent delivery services has been impeded by a number of factors, including behavioral, cultural, and economic ones. These factors include difficulties gaining access to healthcare facilities, insufficient infrastructure, and a lack of skilled personnel for community-based healthcare (14, 22–25). A number of scholars have also clarified why developing countries do not adopt health facility delivery (26–31). Lowering maternal and infant deaths requires first identifying the factors that affect the use of facility-based deliveries. Given the high MMR in low- and middle-income nations, it can help develop interventions and modify policies for important groups in order to enhance the health of mothers and children (32).

An ambitious goal to improve institutional deliveries by 60% and decrease maternal mortality by 75% has been set by Somaliland's Ministry of Health, a major player in the field of maternal and child health. The government has been working hard to promote mother health, but over the last five years, there has been no improvement in the indicators of maternal mortality and morbidity. Moreover, the 2020 Central Statistics Department report states that Somaliland has the third-highest maternal mortality rate in the world, with 396 deaths for every 100,000 live births. Nonetheless, further investigation is required to explore Somaliland women's choices with respect to their birthplace (33, 34).

The World Health Organization's Regional Office (WHO-EMRO), in partnership with the WHO Country Office, University of Aberdeen, and Data and Research Solutions (DARS), conducted a verbal autopsy survey in 2014 and found that the Maternal Mortality Rate (MMR) in Somaliland that was 418 deaths per 100,000 live births but after conducting the first ever Demographic Survey the level of MMR had decreased from 418 deaths per 100,000 live births to 396 deaths in the year of 2020, first official MMR number from the Somaliland Health and Demographic Survey (SLHDS) was made public as a result of the Ministry of Health's disapproval of the report (34, 35).

The authors’ analysis suggests that additional information is required regarding the factors influencing Somaliland's use of institutional delivery services. In addition to offering insightful information, this study, which examined the variables influencing the use of institutional delivery services by women in Somaliland who are of reproductive age, opens the door for further research in this area and encourages advancements in the field.

## Material and methods

### Study area

This research was carried out in East Africa, particularly Somaliland. Geographically, Somaliland is situated next to the Gulf of Aden in the north, Ethiopia in the southwest, Djibouti in the northwest, and Somalia in the east. With a land area of 176,119.2 km^2^, the nation enjoys a temperate climate with dry and wet spells. Six geopolitical regions comprise Somaliland: Awdal, Marodijeh, Sahil, Togdheer, Sanaag, and Sool. An estimated 4.2 million people are living in the nation, the majority of whom are members of Somali ethnic groups that follow Islam. After declaring independence, Somaliland experienced some economic success, but overall, the country made very modest economic improvements. Somaliland's prospects for foreign help and investment have been severely impacted by its lack of international recognition as a sovereign state (36).

### Study design and study period of health demographic survey

Somaliland Health Demographic Survey (SLHDS) was a cross-sectional study the survey conducted in 2019 and reported as the 2020 Somaliland Demographic Health Service (SLDHS). (SLDHS) is secondary data collected from Four distinct questionnaires were employed in 2020, the Maternal Mortality, Household, Ever-married and Never-married Questionnaire. SLDHS was a nationally representative survey that provided information on the health and demographic features of Somaliland's population (37–42).

### Sample size and sampling of health and demographic survey

The current study analyzed data from 3804 women aged 15–49 from the SLHDS dataset. The study utilized a stratified sampling approach, considering six geographic regions and the participants’ residences (urban, rural, or nomadic). To select the enumeration areas (EA) for urban and rural residents, Geographic Information System (GIS) software was employed. The sampling frame comprised 2,806 dwelling structures, including 1,869 urban and 937 rural areas. The selection of the 35 EAs was based on the proportion of the size of the dwelling structures using the probability proportion to size. Subsequently, 10 primary sampling units (PSU) were chosen from the 35 EAs using probability proportion sampling. To construct a sampling frame for nomadic residents, a list of temporary nomadic settlements (TNS) was used as the sampling frame, with the estimated number of households in each TNS serving as the measure of size. A total of 1,448 TNS dwelling structures were identified, and the selection of EAs followed the same process as for urban and rural residents. Finally, a systematic sampling technique was used to select the final sampling units (households) (34).

### Study variables

#### Outcome variable

This study investigated health facility delivery practices among pregnant women in Somaliland, utilizing data from the Somaliland DHS 2020. Although the DHS data did not directly capture health facility delivery practices, the study analyzed responses regarding the place of delivery collected in the SLDHS 2020. The available options for place of delivery included her home, other homes, different governmental health facilities, different types of private health facilities, and others. The study categorizes places of delivery into two groups: health institutions and homes. This study focuses on predicting the place of delivery based on individual- and community-level factors, specifically health facility delivery. The outcome variable is coded as 0 if a woman delivers at home and one if delivery occurs at a health institution.

#### Explanatory variables

In this paper, based on literature (3, 13, 14, 18, 43, 44) the correlates of health facility delivery practices were grouped into two categories, including individual-level and community-level variables, including maternal age group, maternal education level, maternal occupation, husband education level, husband occupation, contraceptive use and intention total children ever born and pregnancy wanted second, the study summarized the literature covering community-level factors including region, place of residence, getting medical help for self: distance to health facility, and household wealth status.

### Data analysis method

All statistical analyses were performed using Stata 17 software. The analysis began with generating a proportion table for the dependent variable (DV), with confidence intervals to assess distribution. This was followed by univariate analysis to examine the frequencies and percentages of all predictor variables. Bivariate analysis, using Chi-square tests, was then conducted to explore associations between the DV and independent variables (IVs). Subsequently, multilevel binary logistic regression was used to assess the impact of both individual- and community-level factors across four models: Model 0: A baseline model containing only the DV. Model I: Includes individual-level IVs. Model II: Focuses solely on community-level variables. Model III: A comprehensive model incorporating both individual and community-level variables. To compare these models, several evaluation metrics were applied: Akaike Information Criterion (AIC): Assesses model fit, penalizing for model complexity. A lower AIC indicates better fit. Bayesian Information Criterion (BIC): Similar to AIC but applies a stricter penalty for model complexity, favoring simpler models. Intraclass Correlation Coefficient (ICC): Reflects the variance explained by group-level differences, with higher ICC values indicating stronger group effects. Log-Likelihood: A higher (less negative) value suggests a better fit, showing how well the model explains the data. Variance: Indicates unexplained variability in the outcome, with lower values representing a better model. A significance threshold of 0.05 was applied throughout the analysis.

## Results

### Magnitude of delivery location

As shown in Table 1, place of delivery” was categorized into two levels: “Home” and “Institutional.” The proportion of deliveries taken place in health institution is 30.8% [95% CI (29.3%–30.2%)], with a standard error of 0.007. In contrast, the proportion of deliveries taken place in home was 69.1% [95% CI (67.7%–70.6%)], also with a standard error of 0.007. This indicates that the vast majority of deliveries taken place in home rather than health institution.

**Table 1:** Magnitude of institutional delivery Among somaliland mothers.

Variable	Levels	Proportion	SE	95% CI
Place of delivery	Home	0.691	0.007	0.677–0.706
	Health Institution	0.308	0.007	0.293–0.322

SE, standard error; CI, confidence interval.

This study, utilizing data from the 2020 Somaliland Demographic and Health Survey (SDHS), reveals significant socio-demographic disparities in the choice of delivery location among women. Age plays a significant role, with younger women aged 15–19 demonstrating the lowest rate of health facility deliveries at 39.74%, compared to the highest observed in women aged 40–44 at 77.03% (*χ*² = 27.326, df = 6, *p* < 0.001). This trend underscores potential barriers faced by younger women in accessing healthcare during childbirth. Education emerges as a powerful determinant; women with no formal education have a markedly low rate of institutional delivery (25.07%), whereas those with higher education show a substantial preference for health facility deliveries (87.72%), a significant difference (*χ*² = 328.534, df = 3, *p* < 0.001). The influence extends to male partners as well, with women whose husbands had some education showing significantly higher rates of health facility deliveries (76.88%) compared to those whose husbands had no education (57.23%) (*χ*² = 362.669, df = 1, *p* < 0.001). Furthermore, economic factors play a crucial role; women from the lowest wealth quintile overwhelmingly opted for home deliveries at 92.61%, while those in the highest wealth quintile show a preference for health facility deliveries at 60.32% (*χ*² = 787.937, df = 1, *p* < 0.001).

Additionally, regional disparities further accentuate these differences in delivery practices. Woqooyi-Galbeed stands out with a higher proportion of women delivering in health facilities at 76.83% whereas Sool demonstrated the lowest rate of health facility deliveries at 64.94% (*χ*² = 50.760, df = 4, *p* < 0.001). This indicates that access to healthcare and societal preferences may vary across regions within Somaliland. Parity also has an impact; women with five or more children are more likely to deliver at home at 75.06% compared to 62.97% among those with less than five (*χ*² = 65.227, df = 1, *p* < 0.001), suggesting that multiple prior births can reduce the likelihood of choosing institutional care. It's noteworthy that neither the place of residence (rural, urban, or nomadic) nor the mother's employment status in the past year had a statistically significant impact on the place of delivery decision (*p* = 0.638 and *p* = 0.803 respectively). However, women who had experience getting medical help showed a greater tendency for health facility deliveries at 73.10% compared to 61.44% among those who did not (*χ*² = 54.075, df = 1, *p* < 0.001), suggesting an influence of previous healthcare experience. Finally, contraceptive use appears to have an influence, women using modern contraceptives are most likely to deliver in a health facility at 60.83% (*χ*² = 96.287, df = 3, *p* < 0.001). Therefore, the findings of this study underscore a clear need for targeted interventions to address disparities in access to safe delivery services (Table 2).

**Table 2:** Socio-Demographic correlates of delivery place Among women in somaliland using 2020 SDHS data.

Variable	Levels	Frequency (*n*) percentage (%)	Place of delivery		Chi-square	Df	*P*-value
			Home (%)	Health institution (%)			
Age in 5-year groups	15–19	156 (4.10)	94 (60.26)	62 (39.74)	27.326	6	0.000
	20–24	763 (20.06)	526 (68.94)	237 (31.06)			
	25–29	1,146 (30.13)	779 (67.98)	367 (32.02)			
	30–34	813 (21.37)	540 (66.42)	273 (33.58)			
	35–39	641 (16.85)	469 (73.17)	172 (26.83)			
	40–44	222 (5.84)	171 (77.03)	51 (22.97)			
	45–49	63 (1.66)	53 (84.13)	10 (15.87)			
Respondent's highest education level	No education	3,159 (83.04)	2,367 (74.93)	792 (25.07)	328.534	3	0.000
	Primary	456 (11.99)	219 (48.03)	237 (51.97)			
	Secondary	132 (3.47)	39 (29.55)	93 (70.45)			
	Higher	57 (1.50)	7 (12.28)	50 (87.72)			
Respondent worked in the last 12 months	Yes	43 (1.13)	29 (67.44)	14 (32.56)	0.062	1	0.803
	No	3,761 (98.87)	2,603 (69.21)	1,158 (30.79)			
Husband ever attended school	No	858 (22.56)	367 (42.77)	491 (57.23)	362.669	1	0.000
	Yes	2,946 (77.44)	2,265 (76.88)	681 (23.12)			
Husband worked in last 12 months	No	1,889 (49.66)	1,521 (80.52)	368 (19.48)	225.898	1	0.000
	Yes	1,915 (50.34)	1,111 (58.02)	804 (41.98)			
Region	Awdal	502 (13.20)	341 (67.93)	161 (32.07)	50.760	4	0.000
	Woqooyi Galbeed	777 (20.43)	597 (76.83)	180 (23.17)			
	Togdheer	789 (20.74)	494 (62.61)	295 (37.39)			
	Sool	830 (21.82)	539 (64.94)	291 (35.06)			
	Sanaag	906 (23.82)	661 (72.96)	245 (27.04)			
Type of place of residence	Rural	1,204 (31.65)	822 (68.27)	382 (31.73)	0.899	2	0.638
	Urban	1,128 (29.65)	780 (69.15)	348 (30.85)			
	Nomadic	1,472 (38.70)	1,030 (69.97)	442 (30.03)			
Wealth index	Lowest	1,069 (28.10)	990 (92.61)	79 (7.39)	787.937	1	0.000
	Second	516 (13.56)	436 (84.50)	80 (15.50)			
	Middle	546 (14.35)	421 (77.11)	125 (22.89)			
	Fourth	748 (19.66)	418 (55.88)	330 (44.12)			
	Highest	925 (24.32)	367 (39.68)	558 (60.32)			
Total children ever born	Less than 5	1,847 (48.55)	1,163 (62.97)	684 (37.03)	65.227	1	0.000
	Five and more	1,957 (51.45)	1,469 (75.06)	488 (24.94)			
Getting Medical help for self: distance to health facility	Yes	2,528 (66.46)	1,848 (73.10)	680 (26.90)	54.075	1	0.000
	No	1,276 (33.54)	784 (61.44)	492 (38.56)			
Contraceptive use and intention	Using modern method	120 (3.15)	47 (39.17)	73 (60.83)	96.287	3	0.000
	Using traditional method	4 (0.11)	3 (75.00)	1 (25.00)			
	Non-User intends to use later	386 (10.15)	214 (55.44)	172 (44.56)			
	Does not intend to use	3,294 (86.59)	2,368 (71.89)	926 (28.11)			
Wanted pregnancy when I became pregnant	Then	2,861 (75.21)	1,961 (68.54)	900 (31.46)	4.174	2	0.124
	Later	786 (20.66)	552 (70.23)	234 (29.77)			
	No more	157 (4.13)	119 (75.80)	38 (24.20)			

This study, employing a multilevel analysis on 2020 Somaliland DHS data, reveals the complex interplay of individual and community-level factors influencing women's decisions regarding delivery location. The empty model (Model I) demonstrates that a substantial 50% of the variance in institutional delivery is attributable to community-level factors. Model II, focusing on individual-level variables, reveals that education is a crucial determinant. Women with primary education were 1.78 times more likely (AOR = 1.78, *p* < 0.05) to deliver in a health facility compared to those with no education, with this likelihood increasing to 2.27 for secondary education and a significant 9.31 for higher education (*p* < 0.05). Conversely, women whose husbands had attended school were significantly less likely to opt for institutional delivery (AOR = 0.44, *p* < 0.05), while those whose husbands were employed had higher odds of facility delivery (AOR = 1.33, *p* < 0.05). Additionally, women with five or more children were significantly less likely to deliver at a health institution (AOR = 0.44, *p* < 0.05), and those who did not intend to use contraceptives were also less likely to choose institutional delivery (AOR = 0.40, *p* < 0.05). Furthermore, women who wanted their pregnancy later were significantly less likely to deliver at health facilities (AOR = 0.74, *p* < 0.05).

Geographic and socioeconomic influences on delivery location findings also show when considering community-level variables in Model III, notable disparities emerged. Women in Waqooyi-Galbeed (AOR = 0.54, *p* < 0.05) and Sanaag (AOR = 0.40, *p* < 0.05) regions showed significantly lower odds of institutional delivery compared to those in Awdal region. Nomadic populations were also significantly less likely to have institutional deliveries than those in rural settings (AOR = 0.26, *p* < 0.05). The wealth index emerged as a powerful predictor, with women in the highest wealth quintile being over 31 times more likely to deliver in a health facility compared to those in the lowest quintile (AOR = 31.34, *p* < 0.05). Notably, accessing medical help for self-did not significantly impact the choice of place for delivery. In Model IV, which combined both individual and community-level factors, some effects persisted but attenuated. The positive impact of education persisted as a strong determinant (AOR = 8.87 for higher education, *p* < 0.05). The negative effects of residing in Sanaag region (AOR = 0.36, *p* < 0.05) and in a nomadic setting (AOR = 0.28, *p* < 0.05) also persisted, emphasizing the influence of place of residence, while the wealth index still played an important role, with women in the highest wealth quintile being almost 19 times more likely to deliver at a health facility (AOR = 18.71, *p* < 0.05). Similarly, multiparous women (AOR = 0.52, *p* < 0.05) and those who do not intend to use contraceptives (AOR = 0.62, *p* < 0.05) were still significantly less likely to deliver in health facilities, with those who wanted pregnancy later also less likely to deliver at a health facility (AOR = 0.79, *p* < 0.05). The significant reduction in the Intraclass Correlation Coefficient (ICC) from 50% in Model I to 18.3% in Model IV, alongside decreasing AIC and BIC values, highlights the improvement in model fit, yet indicates that unobserved community-level factors continue to influence delivery choices.

Therefore, these findings underscore the need for multifaceted interventions to promote institutional delivery in Somaliland. While individual factors like education for women are crucial, the impact of community factors including wealth, region of residence and place of residence are significant. There is a clear need for regionally targeted strategies and interventions that overcome the challenges faced by nomadic populations. Wealth also has a persisting effect with a clear need for policies aimed at reducing the socioeconomic disparities. The strong positive effect of women's education highlights the need for education initiatives targeted to women. Furthermore, parity and intention to use contraceptives were a significant determinant of where women give birth, also pointing towards a need to address these in interventions. By tackling these interwoven factors, it is possible to effectively reduce disparities and promote safer delivery practices across Somaliland (Table 3).

**Table 3:** Multilevel analysis of determinants of institutional delivery among women in Somaliland using SDHS 2020 data.

Variable	Levels	Model I (empty model)	Model II (individual level variables)	Model III (community level variables)	Model IV (all variables)
		AOR (95% CI) ^a^*P*-value	AOR (95% CI) ^a^*P*-value	AOR (95% CI) ^a^*P*-value	AOR (95% CI) ^a^*P*-value
Age in 5-year groups	15–19		1		1
	20–24		0.60 (0.38–0.93)^a^		0.65 (0.41–1.02)
	25–29		0.78 (0.50–1.20)		0.71 (0.45–1.12)
	30–34		1.13 (0.71–1.80)		0.99 (0.61–1.60)
	35–39		1.06 (0.65–1.73)		0.99 (0.59–1.64)
	40–44		1.21 (0.67–2.16)		0.84 (0.45–1.54)
	45–49		0.53 (0.21–1.32)		0.41 (0.16–1.06)
Respondent's highest education level	No education		1		1
	Primary		1.78 (1.38–2.30)^a^		1.39 (1.07–1.80)^a^
	Secondary		2.27 (1.41–3.63)^a^		2.30 (1.44–3.68)^a^
	Higher		9.31 (3.51 24.66)^a^		8.87 (3.47–22.65)^a^
Respondent worked in the last 12 months	Yes		1		1
	No		0.93 (0.39 2.19)		0.83 (0.34 2.04)
Husband ever attended school	No		1		1
	Yes		0.44 (0.35–0.54)^a^		0.56 (0.45–0.70)
Husband worked in last 12 months	No		1		1
	Yes		1.33 (1.09–1.62)^a^		1.07 (0.88–1.32)
Region	Awdal			1	1
	Woqooyi Galbeed			0.54 (0.36–0.80)^a^	0.53 (0.35–0.81)^a^
	Togdheer			0.91 (0.61–1.34)	0.93 (0.62–1.40)
	Sool			0.77 (0.51–1.15)	0.72 (0.47–1.09)
	Sanaag			0.40 (0.28-0.59)^a^	0.36 (0.24–0.54)^a^
Type of place of residence	Rural			1	1
	Urban			1.12 (0.84–1.47)	1.07 (0.81–1.43)
	Nomadic			0.26 (0.19-0.36)^a^	0.28 (0.20-.38)^a^
Wealth index	Lowest				1
	Second			3.33 (2.21–5.03) ^a^	3.18 (2.09–4.85) ^a^
	Middle			6.77 (4.46–10.26) ^a^	5.84 (3.79–9.02) ^a^
	Fourth			16.83 (11.31–25.06) ^a^	12.72 (8.34–19.39) ^a^
	Highest			31.34 (21.06–46.65) ^a^	18.71 (12.17–28.78) ^a^
Total children ever born	Less than 5		1		1
	Five and more		0.44 (0.35–0.55)^a^		0.52 (0.41–0.65)^a^
Contraceptive use and intention	Using modern method		1		1
	Using traditional method		0.08 (0.005–1.38)		0.14 (0.008–2.44)
	Non-User intends to use later		0.71 (0.42–1.21)		0.96 (0.56–1.64)
	Does not intend to use		0.40 (0.24–0.65)^a^		0.62 (0.38–1.008)
Getting Medical help for self: distance to health facility	Yes			1	1
	No			0.96 (0.80 1.16)	0.85 (0.70–1.04)
Wanted pregnancy when became pregnant	Then		1		1
	Later		0.74 (0.60–93)^a^		0.79 (0.63–0.99)^a^
	No more		0.69 (0.43–1.12)		0.67 (0.41–1.09)
Random effects					
ICC (%)		.5007242	.4070703	.1825567	.1830133
Standard Error		.0411105	.0431583	.0332741	.0347932
Model Statistics					
AIC		3,942.031	3,694.377	3,567.104	3,440.165
BIC		3,954.519	3,810.543	3,648.273	3,633.723

AOR, adjusted odds ratio; CI, confidence interval, ^a^significance level of <0.5.

## Discussion

This study, titled “Factors associated with institutional delivery service utilization among women of childbearing age in Somaliland using 2020 Somaliland Demographic Health Survey,” provides crucial insights into the determinants of institutional delivery in a setting characterized by high maternal mortality rates. Our analysis of the 2020 SDHS data reveals that a substantial 69.1% of deliveries occur at home, highlighting the critical need for interventions aimed at promoting facility-based births. This finding aligns with the broader context of low-income countries, as highlighted in the introduction, where institutional delivery rates are often low, contributing to higher maternal and newborn mortality (1, 2, 6–8). Our study provides a detailed exploration of the specific factors within Somaliland that contribute to this issue.

The existing literature emphasizes the importance of institutional delivery for reducing maternal mortality, which is also a point reinforced by our study's findings. Like other studies mentioned (4, 17–19), we find a significant association between socio-economic factors and the use of institutional delivery. For instance, our study corroborates findings from previous research (11–14) by identifying poverty as a major barrier to health facility deliveries. Women from the lowest wealth quintile were significantly less likely to deliver at a health institution compared to their wealthier counterparts. Similarly, our findings on the positive association between maternal education and institutional delivery rates are consistent with prior literature (26–31), emphasizing the importance of empowering women through education. The study also shows regional differences in rates of health facility deliveries, which is in line with the prior understanding that access and utilization of health facilities varies regionally (11). Moreover, the study also supports the previous literature on the role of individual factors such as parity and intention to use contraceptives on the choice of place of delivery, highlighting a strong link between healthcare-seeking behaviors. Similar to previous research (14, 22–25), we also found that the presence of medical help for self does not significantly impact the choice of place for delivery suggesting that distance may not always be the limiting factor for choice of delivery site.

While many of our findings align with existing literature, our study also offers novel contributions specific to the Somaliland context. Unlike some studies that focus solely on individual-level determinants, we employed a multilevel analysis that accounted for both individual and community-level factors. Our study reveals that community-level factors account for 50% of the variation in place of delivery (Model I), highlighting that where a woman lives significantly influences her delivery choices. This is a unique finding and an important contribution to the existing literature. Furthermore, the finding that living in a nomadic setting significantly decreases the odds of institutional delivery is particularly relevant to Somaliland, where nomadic populations constitute a substantial portion of the population. While previous literature has highlighted barriers like distance and lack of infrastructure as limitations, our study shows that residing in a nomadic community is an independent risk factor even after controlling for other socioeconomic factors. Interestingly, our study found an inverse association between the husband's education and institutional delivery, which contrasts with much of the previous literature where male partners’ education and employment was an enabler. While in model II the husband's education is significantly negatively associated with the choice of institutional delivery, the effect of husband's education becomes insignificant in the full model (Model IV), which is a novel finding specific to this context. Our study also shows that women who wanted their pregnancy later were less likely to deliver at a health facility, an observation not widely documented in literature, suggesting a need for further investigation in this area.

### Implications and policy recommendations

The results of this study carry significant implications for policy and interventions in Somaliland. The high prevalence of home births (69.1%) and the identified factors call for a strategic, multi-faceted approach. The study adds to existing literature by pointing out that improving health outcomes in Somaliland requires a simultaneous focus on individual and community-level factors, with special attention to the needs of specific vulnerable communities. For instance, interventions should not only target women directly but also address broader community-level factors such as infrastructure in nomadic communities and regional disparities by providing targeted services in areas like Waqooyi-Galbeed and Sanaag. The strong effect of women's education on health outcomes highlights the need to target investment on women's education, while wealth disparities can be overcome through targeted interventions for the most vulnerable. Furthermore, interventions should address the underlying causes of high parity and low contraceptive use by providing better family planning services. The study's contribution in the local context is the recommendation for Somaliland's health planners to adopt targeted interventions addressing regional disparities, nomadic communities, low education levels, wealth disparity, parity, low contraception uptake, and lack of maternal support systems.

## Conclusion

In conclusion, this study provides a comprehensive analysis of the factors influencing institutional delivery in Somaliland. Our results align with existing literature on the importance of socio-economic factors, yet make unique contributions by highlighting the significance of community-level factors, the importance of place of residence (nomadic) and the influence of parity and contraceptive use intention. These findings highlight the complexities of health-seeking behaviors within the Somaliland context. Ultimately, our work underscores the need for targeted, context-specific interventions that address both individual and community-level factors in order to improve maternal health outcomes in Somaliland.

## Data Availability

The original contributions presented in the study are included in the article/Supplementary Material, further inquiries can be directed to the corresponding author.
